# Application of high-flow nasal cannula in hypoxemic patients with COVID-19: a retrospective cohort study

**DOI:** 10.1186/s12890-020-01354-w

**Published:** 2020-12-24

**Authors:** Ming Hu, Qiang Zhou, Ruiqiang Zheng, Xuyan Li, Jianmin Ling, Yumei Chen, Jing Jia, Cuihong Xie

**Affiliations:** 1 Department of Critical Care Medicine, Wuhan Pulmonary Hospital, Wuhan, 430030 China; 2 Division of Cardiology, Department of Internal Medicine, Tongji Hospital, Tongji Medical College, Huazhong University of Science and Technology, Wuhan, 430030 China; 3 Department of Critical Care Medicine, Northern Jiangsu People’s Hospital, Yangzhou, 225001 Jiangsu Province China; 4 Department of Respiratory and Critical Care Medicine, Beijing Institute of Respiratory Medicine, Beijing Chao-Yang Hospital, Capital Medical University, Beijing, 100020 China; 5 Department of Emergency and Critical Care Medicine, Tongji Hospital, Tongji Medical College, Huazhong University of Science and Technology, Wuhan, 430030 China

**Keywords:** COVID-19, high-flow nasal cannula oxygen therapy, predictive factor, ROX index, respiratory support

## Abstract

**Background:**

It had been shown that High-flow nasal cannula (HFNC) is an effective initial support strategy for patients with acute respiratory failure. However, the efficacy of HFNC for patients with COVID-19 has not been established. This study was performed to assess the efficacy of HFNC for patients with COVID-19 and describe early predictors of HFNC treatment success in order to develop a prediction tool that accurately identifies the need for upgrade respiratory support therapy.

**Methods:**

We retrospectively reviewed the medical records of patients with COVID-19 treated by HFNC in respiratory wards of 2 hospitals in Wuhan between 1 January and 1 March 2020. Overall clinical outcomes, the success rate of HFNC strategy and related respiratory variables were evaluated.

**Results:**

A total of 105 patients were analyzed. Of these, 65 patients (61.9%) showed improved oxygenation and were successfully withdrawn from HFNC. The PaO_2_/FiO_2_ ratio, SpO_2_/FiO_2_ ratio and ROX index (SpO_2_/FiO_2_*RR) at 6h, 12h and 24h of HFNC initiation were closely related to the prognosis. The ROX index after 6h of HFNC initiation (AUROC, 0.798) had good predictive capacity for outcomes of HFNC. In the multivariate logistic regression analysis, young age, gender of female, and lower SOFA score all have predictive value, while a ROX index greater than 5.55 at 6 h after initiation was significantly associated with HFNC success (OR, 17.821; 95% CI, 3.741-84.903 *p*<0.001).

**Conclusions:**

Our study indicated that HFNC was an effective way of respiratory support in the treatment of COVID-19 patients. The ROX index after 6h after initiating HFNC had good predictive capacity for HFNC outcomes.

## Background

In 2020, COVID-19 swept across the world and has caused nearly 300,000 deaths. The main clinical manifestations of COVID-19 are fever, cough, and pneumonia characterized by patchy and ground glass opacities on chest imaging [1]. Severe patients can develop ARDS and progress to acute respiratory failure leading to death. Most of the current reports found that the mortality rate of severe COVID-19 patients was high, and most of the patients died of severe hypoxemia [2, 3]. Therefore, it is essential for respiratory support therapy in patients with severe COVID-19. At present, there is still some controversy about the respiratory support treatment of COVID-19 in clinical practice. There is no clear conclusion about the indication of noninvasive respiratory support and when tracheal intubation is needed.

Although the clinical application of High-flow nasal cannula oxygen therapy (HFNC) is not long, many studies have confirmed that the use of HFNC in patients with acute respiratory failure (ARF) is safe and effective [4, 5]. One large randomized control trial comparing the effectiveness of conventional oxygen therapy, noninvasive ventilation (NIV) combined with HFNC, and HFNC alone in hypoxemic ARF demonstrated that HFNC alone reduced need for invasive mechanical ventilation (IMV) in the most severe (PaO_2_/FiO_2_, ≤200 mm Hg) subgroup of patients. HFNC patients also had the higher 90-day survival rate of the entire cohort [6]. Therefore, the use of HFNC in acute respiratory failure is widely accepted, and in this outbreak of COVID-19, HFNC is also widely used in patients with severe COVID-19.

However, one of the most challenging decisions in the management of ARF patients is to decide when to move from a spontaneous breathing oxygenation therapy to IMV [7]. This is also a concern when using HFNC to treat patients with COVID-19. In this regard, although HFNC may avoid further need for IMV in some patients [8, 9], it may unduly delay initiation of IMV in others and worsen their outcome [10], as already evidenced for NIV [11, 12]. Therefore, to identify and describe accurate early predictors of the need for IMV in spontaneously breathing patients are of special interest. WHO has also pointed out that the oxygenation status of COVID-19 patients should be closely monitored when using HFNC in order to timely adjust the respiratory support program.

Some indicators have been shown to be useful in monitoring oxygenation status in patients with HFNC and in predicting the outcome of HFNC. Oxygen saturation index (SpO_2_/FiO_2_) and respiratory rate-oxygenation index (ROX index: SpO_2_/FiO_2_*RR) have been reported to be an effective monitoring indicator in the application of HFNC [13–15]. However, it is not known whether these indicators are still applicable in COVID-19 patients.

In this study, we retrospectively analyzed the efficacy of HFNC in COVID-19 patients with hypoxic respiratory failure and the predictive values of SpO_2_/FiO_2_ and ROX index in terms of HFNC outcomes.

## Methods

### Study design and patients

This was retrospective observational study in which all cases were collected from respiratory wards of two hospitals in Wuhan during COVID-19 outbreak. All data were extracted from clinical records. The retrospective data analysis was approved by the ethics board of Wuhan Pulmonary Hospital and Tongji Hospital Affiliated to Tongji Medical College, Huazhong University of Science and Technology. The need for patient consent was waived because of the retrospective nature of the study.

All patients initially admitted to the respiratory department instead of ICU and treated with HFNC (AIRVO2, Fisher&Paykel Healthcare) were included between 1 January and 1 March 2020. COVID-19 was diagnosed according to diagnosis and clinical classification criteria and treatment plan (trial version 7) of the SARS-CoV-2 coronavirus pneumonia (COVID-19) launched by the National Health Committee of the People's Republic of China. Exclusion criteria were age younger than 18 years and indication for immediate IMV [16] upon admission.

### Study variables

Demographic, clinical, laboratory, management, and outcome data were obtained from the medical records. Respiratory rate and pulmonary gas exchange variables were recorded 0, 2, 6, 12, and 24 hours after initiation of HFNC therapy. After the first 24 hours, the same variables were recorded once daily until HFNC withdrawal. The presence of an organ failure before and during HFNC therapy was also registered. Briefly, shock was defined as need for vasopressors; renal failure was defined as increased serum creatinine × 1.5 and/or urine output less than 0.5 mL/kg per hour during 6 hours. Length of HFNC therapy and hospital stay were also investigated. The outcome measures were the success rates of HFNC and overall survival after initiating HFNC. Successful HFNC treatment was defined as HFNC withdrawal with improved oxygenation, no need for NIV and/or IMV, discharge alive. HFNC failure was defined as the need for NIV or IMV and/or death while on HFNC support.

### HFNC treatment strategy

HFNC indications: Patients with SpO2≤92% and / or RR≥25 times/min under nasal tube oxygen inhalation 10L/min or mask oxygen supply. HFNC settings: The initial HFNC set the gas flow rate to 30L/min and the FiO2 of 1.0, adjust the flow rate and FiO2 to maintain the pulse oxygen saturation (SpO2) at 92%-96%, and dynamically adjust it based on the blood gas analysis results.

### Statistical analysis

We summarized the patients’ baseline characteristics using percentages for categorical variables and medians and interquartile ranges for continuous variables. The nonparametric Mann–Whitney U test was used to analyze continuous variables, and Fisher’s exact test was used for categorical variables. To assess the accuracy of different variables for correctly classifying patients who would succeed or fail on HFNC, receiver operating characteristic (ROC) curves were performed, and the areas under the ROC curve (AUROC) were calculated. The optimal cutoff point of continuous variables was chosen to maximize the sum of the sensitivity and specificity. Multivariate analysis was performed using logistic regression analysis to identify independent predictive factors for HFNC success or failure. Factors with a p value less than 0.10 in the univariable analyses were included in the multivariate model. The significance level was defined as p<0.05. All statistical analyses were performed using SPSS, version 17.0.

## Results

### Clinical characteristics of included patients

**Fig. 1:**
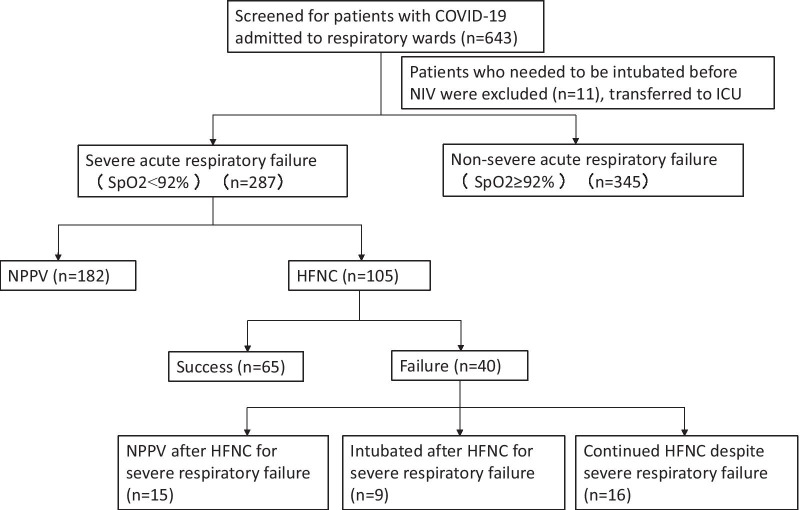
Diagram of patients flow in this study. Non-invasive ventilation, NIV; Non-invasive positive pressure ventilation, NPPV; HFNC, high-flow nasal cannula oxygen therapy

During the study period, 105 patients with severe COVID-19 were treated with HFNC (Fig. 1). The demographics of the study population are shown in Table 1. The patients comprised 51 men and 54 women with a median age of 64 years. The average age of HFNC failure group was significantly higher than that of HFNC success group (*p* < 0.001). Overall, 11 (10.5%) patients had a history of smoking and 60 (57.1%) had comorbidities, with hypertension being the most common. Laboratory tests revealed that all patients had decreased lymphocyte counts, elevated CRP, and elevated D-dimers. The median PaO_2_/FiO_2_ ratio at HFNC application was 116. The HFNC failure patients had a higher PSI score, APACHE II score and SOFA score (*p*< 0.001, *p*= 0.006, *p*< 0.001, respectively). Of all the patients, 65 patients (61.9%) showed improved oxygenation and were successfully withdrawn from HFNC. Of the 40 patients for whom HFNC treatment failed, 15 were switched to NPPV, 9 were switched to IMV, and 16 continued HFNC until death (Fig. 1). The two main reasons for the last part were that some family members refused to tracheal intubation and some patients could not tolerate NPPV. The median duration of HFNC therapy and hospitalization were 6.8 days and 14 days.

**Table 1:** Characteristics of patients with severe COVID-19 treated with HFNC

Characteristics	All patients (*n* = 105)	Outcome of HFNC treatment		*p* value
		Success (*n* = 65)	Failure (*n* = 40)	
Baseline characteristics				
Age (years)	64.0±11.3	59.5±10.9	71.3±7.6	<0.001
Sex, male	51 (48.6%)	26 (40.0%)	25 (62.5%)	0.025
Smoking	11 (10.5%)	7 (10.8%)	4 (10.0%)	0.901
Comorbidities	60 (57.1%)	35 (53.8%)	25 (62.5%)	0.384
Lab tests at admission				
LYM (× 10^9^/L; normal range 1.1–3.2)	0.63 (0.43–0.80)	0.62 (0.49–0.79)	0.70 (0.36–0.80)	0.777
D–D (ug/ml; normal range 0.0–0.5)	0.67 (0.42–4.19)	0.62 (0.42–1.78)	1.04 (0.46–5.00)	0.056
CRP (mg/L; normal range 0.0–5.0)	46.8 (28.2–83.5)	45.6 (30.4–83.5)	39.3 (23.4–85.4)	0.946
Time from onset of symptom to hospital admission (days)	10.0 (7.0–12.0)	10.0 (7.0–12.0)	9.0 (5.0–12.0)	0.373
Time from admission to HFNC application (days)	1.0 (0.0–2.0)	1.0 (0.0–2.0)	1.0 (1.0–2.0)	0.129
PaO_2_/FiO_2_ at HFNC application	116.0 (102.1–132.0)	116.0 (102.7–128.0)	112.8 (100.5–138.5)	0.722
PSI	76.0 (54.0–82.5)	62.0 (49.0–80.0)	81.5 (78.0–97.5)	<0.001
APACHE II of 24h admission	8.0 (6.5–10.0)	8.0 (5.0–10.0)	9.0 (8.0–10.8)	0.006
SOFA admission	3.0 (3.0–4.0)	3.0 (3.0–3.0)	4.0 (3.0–5.0)	<0.001
Length of HFNC (days)	5.0 (2.5–9.0)	6.0 (3.5–8.5)	3.0 (2.0–11.0)	0.115
Length of hospital stay (days)	14.0 (10.5–19.0)	14.0 (12.0–20.0)	11.5 (7.0–14.0)	0.001

Each parameter is expressed as number (percentage) or median (interquartile range). Parameters in each group were compared using Fisher’s exact test or the Mann–Whitney U test. HFNC, high-flow nasal cannula oxygen therapy; LYM, lymphocyte number; D-D, D-dimer; CRP, C-reaction protein

### Impact of respiratory variables during treatment on HFNC outcome

All patients were examined for partial pressure of oxygen in blood gas at 2, 6, 12 and 24h after HFNC, and respiratory rates, oxygen concentration, finger oxygen saturation were collected to calculate the PaO_2_/FiO_2_, SpO_2_/FiO_2_, SpO_2_/FiO_2_*RR at each time points. Indicators for 2 patients were collected up to 6h because one patient converted to non-invasive ventilation after 6 h and the other died quickly after 6 h; indicators for 4 patients were collected up to 12h, again because 3 patients converted to noninvasive ventilation and the other incubated. The remaining 99 patients collected indicators at all time points. HFNC success patients had higher SpO_2_/FiO_2_, PaO_2_/FiO_2_ and lower RR at 6,12 and 24h of HFNC onset, respectively (Table 2). Significant differences were observed in ROX index after 6h of HFNC treatment between success and failure HFNC patients (Table 2). The differences increased throughout the study period. The SpO_2_/FiO_2_, PaO_2_/FiO_2_ and ROX index had a same trend, that is, they gradually increased in the HFNC success group, and gradually declined in the HFNC failure group. Their accuracy to predict success of HFNC was assessed by calculating the AUROC (Table 3). None of the variables analyzed at 2h after HFNC had good predictive capacity for outcomes of HFNC (AUROC, <0.7). After 6h of HFNC treatment, SpO_2_/FiO_2_, PaO_2_/FiO_2_ and ROX index demonstrated a good prediction accuracy (AUROC, 0.786, 0.749, 0.798, respectively). Using the ROC curve, the best cutoff point for the ROX index at 6h was estimated to be 5.55. A ROX index greater than 5.55 at 6h after HFNC onset has a sensitivity of 61.1%, a specificity of 84.6%, a positive predictive value of 68.8%, a negative predictive value of 79.8%.

**Table 2:** Changes in respiratory variables during HFNC

Variables	Time (h)	HFNC success	HFNC failure	*P*
RR	2	24 (22–26)	25 (23–27)	0.138
	6	22 (21–24)	24 (23–26)	0.001
	12	22 (20–25)	25 (24–25)	0.002
	24	21 (20–23)	25 (25–28)	<0.001
SpO_2_/FiO_2_	2	153.2 (135.6–194.9)	158.3 (139.8–170.0)	0.157
	6	158.6 (135.3–215.3)	123.8 (116.7–157.9)	<0.001
	12	179.6 (136.1–206.5)	127.0 (115.3–161.7)	<0.001
	24	182.7 (142.8–202.1)	126.4 (116.0–153.8)	<0.001
PaO_2_/FiO_2_	2	116.7 (93.8–143.8)	111.1 (100.0–125.0)	0.141
	6	115.4 (100.8–164.3)	95.3 (83.5–120.3)	<0.001
	12	130.0 (104.6–168.8)	90.7 (76.9–106.3)	<0.001
	24	145.0 (107.2–167.3)	85.2 (72.9–110.9)	<0.001
ROX index	2	6.8 (5.6–7.8)	6.4 (4.9–7.6)	0.074
	6	6.7 (5.9–9.5)	5.0 (4.6–6.5)	<0.001
	12	7.9 (6.1–9.1)	5.0 (4.4–7.3)	<0.001
	24	7.8 (6.6–10.0)	4.8 (4.4–6.0)	<0.001

Each parameter is expressed as median (interquartile range). Parameters in each group were compared using the Mann-Whitney U test. *RR* respiratory rate, *SpO*_*2*_ pulse oxygen saturation, *FiO*_*2*_ fraction of inspired oxygen, *PaO*_*2*_ arterial partial pressure of oxygen, *ROX index* Respiratory rate-oxygenation index

**Table 3:** Decision accuracy of the outcome of high-flow nasal cannula oxygen therapy

	Variable	AUROC	95% CI
2 h	SpO_2_/FiO_2_	0.536	0.421–0.651
	PaO_2_/FiO_2_	0.540	0.426–0.654
	ROX index	0.560	0.444–0.677
6 h	SpO_2_/FiO_2_	0.786	0.693–0.878
	PaO_2_/FiO_2_	0.749	0.648–0.850
	ROX index	0.798	0.703–0.893
12 h	SpO_2_/FiO_2_	0.805	0.717–0.892
	PaO_2_/FiO_2_	0.832	0.751–0.913
	ROX index	0.820	0.727–0.913
24 h	SpO_2_/FiO_2_	0.818	0.732–0.903
	PaO_2_/FiO_2_	0.855	0.780–0.930
	ROX index	0.874	0.799–0.949

*AUROC* area under the receiver operating characteristic curve, *CI* confidence interval, *SpO*_*2*_ pulse oxygen saturation, *FiO*_*2*_ fraction of inspired oxygen, *PaO*_*2*_ arterial partial pressure of oxygen, *ROX* Respiratory rate-oxygenation index

### Univariate and multivariate analyses of predictive factors for HFNC outcome

In the univariate analysis, age, gender, PSI, APACHE II and SOFA were relevant influencing factors for the HFNC success (Table 4). More significantly, a ROX index greater than 5.55 (OR, 8.643; CI, 3.342-22.354; *p*<0.001) at 6h of HFNC application, was significant predictors of HFNC outcome. However, incorporating all the indicators related to HFNC success in univariate analysis into multivariate analysis found that young age, gender of female, SOFA and ROX index were independent prognostic factors of HFNC success. Among these indicators, the ROX index greater than 5.55 at 6h of HFNC application is the most relevant predictor of HFNC success (OR, 17.821; 95% CI, 3.741-84.903; *p*<0.001).

**Table 4:** Univariate and multivariate analyses of predictive factors for successful HFNC

Variable	Odds ratio	95% CI	*p* value
Univariate analysis of predictive factors of the outcome of HFNC			
Age, years	0.871	0.819–0.926	<0.001
Sex, male	0.400	0.178–0.899	0.027
Comorbidities	0.700	0.313–1.565	0.385
Smoking, current or former	1.086	0.297–3.973	0.901
LYM (× 10^9^/ L; normal range 1.1–3.2)	1.045	0.235–4.657	0.954
D–D (ug/ml; normal range 0.0–0.5)	1.001	0.947–1.058	0.974
CRP (mg/L; normal range 0.0–5.0)	0.999	0.988–1.011	0.922
PaO_2_/FiO_2_ at HFNC application	1.002	0.985–1.018	0.830
PSI	0.931	0.903–0.960	<0.001
APACHE-II	0.822	0.710–0.951	0.008
SOFA	0.501	0.338–0.744	0.001
6h ROX index >5.55, yes	8.643	3.342–22.354	<0.001
Multivariate analysis of predictive factors of the outcome of HFNC			
Age, years	0.837	0.745–0.940	0.003
Sex, male	0.172	0.038–0.790	0.024
PSI	1.004	0.939–1.074	0.903
APACHE-II	1.166	0.858–1.585	0.326
SOFA	0.389	0.203–0.745	0.004
6h ROX index >5.55, yes	17.821	3.741–84.903	<0.001

*HFNC* high-flow nasal cannula oxygen therapy, *LYM* lymphocyte number, *D-D*, D-dimer, *CRP* c-reaction protein, *PSI* pneumonia severity index, *APACHE-II* Acute Physiology and Chronic Health Evaluation II, *SOFA* Sepsis-related Organ failure Assessment, *ROX index* Respiratory rate-oxygenation index

## Discussion

The current study was conducted to evaluate the efficacy of HFNC in COVID-19 patients with hypoxic respiratory failure. Our results showed that HFNC was an effective treatment for these patients, and approximately 61.9% of patients showed improved oxygenation and were able to successfully withdraw from HFNC. Furthermore, The PaO_2_/FiO_2_, SpO_2_/FiO_2_ and ROX index after 6h HFNC application can predict the success of HFNC application. The ROX index at 6h HFNC application has best predictive value when considering both statistical and clinical significance.

A typical characteristic of the severe acute respiratory syndrome-related coronavirus-2 (SARS-CoV-2) infected patient is pneumonia, now termed as COVID-19. Generally, the patients showed the acute respiratory infection symptoms, with some that quickly developed acute respiratory failure and even died of refractory hypoxemia [17, 18]. Therefore, respiratory support, especially oxygen therapy, is very important in the treatment of severe COVID-19. However, there is still controversy about whether invasive ventilator treatment or non-invasive ventilator treatment is better for COVID-19 patients, especially there are obvious complications of infection in the late stage of intubation due to the long course of the disease [19]. As a new method of oxygen therapy, HFNC can effectively improve oxygenation, reduce the probability of invasive and non-invasive mechanical ventilation. HFNC provides sufficiently heated and humidified oxygen to relieve nasal cavity irritation. It has obvious advantages over traditional oxygen therapy [20–22]. Previous studies have found that HFNC can be used in ICU patients with acute hypoxemic respiratory failure [4, 23]. HFNC has been reported to be superior to NPPV in terms of both mortality and comfort [24]. However, in COVID-19, pulmonary lesions often begin with interstitial exudation and gradually progress to large consolidation, and lung compliance significantly decreases. In addition to the long course of disease, ventilator-related complications, such as barotrauma and ventilator-related infections, are prone to occur in the mid-term after IMV treatment. Therefore, the use of HFNC in COVID-19 has certain advantages. More than half of the patients in our study eventually successfully weaned from the ventilator, suggesting that HFNC is a treatment worth considering for COVID-19. In addition, we found that although there was no significant difference in the oxygenation index between HFNC success group and HFNC failure group at the beginning of HFNC treatment, PSI, APACHII and SOFA were significantly lower in the survival group than that in the death group, which suggesting that the patients with successful HFNC treatment were relatively mild. Accordingly, multivariate regression analysis found that young age, gender of female, and lower SOFA were independent prognostic factors of the outcome of HFNC. It means that the treatment strategy of HFNC needs to be determined in the context of the overall severity of the patients with COVID-19.

During the treatment of HFNC, how to judge the therapeutic effect, when HFNC should continue, and when HFNC needs to be converted to IMV or NPPV have always been a concern. In particular, studies have found that delayed intubation in HFNC may lead to increased mortality [25]. Therefore, how to determine the poor therapeutic effect of HFNC in the early stage and timely change the ventilator support mode is the most critical issue in the use of HFNC. PaO_2_/FiO_2_ has been the gold standard for judging patients' oxygenation status. In our study, we also found that the level of PaO_2_/FiO_2_ after 6h of HFNC was significantly correlated with the outcome of treatment (AUROC > 0.75). However, the acquisition of PaO_2_/FiO_2_ needs to draw patients' arterial blood regularly, which is not easy to implement sometimes.

The relationship between SpO_2_/FiO_2_ and PaO_2_/FiO_2_ is linear and can be described by the following equation: SpO_2_/FiO_2_ = 64 + 0.84*(PaO_2_/FiO_2_) [26]. Studies have found that ARDS patients diagnosed by SpO_2_/FiO_2_ and PaO_2_/FiO_2_ have similar clinical characteristics and prognosis [27]. SpO_2_/FiO_2_ and PaO_2_/FiO_2_ correlated well in our study, and SpO_2_/FiO_2_ was clearly correlated with prognosis after 6h of HFNC application (AUROC ≈ 0.8). ROX index is an index of the effect of respiratory rate added to SpO_2_/FiO_2_. From the results of this study in COVID-19 patients with respiratory failure, the predicted value of the ROX index is relatively higher than the SpO_2_/FiO_2_. Oriol et al. [28] have reported ROX index greater than 4.88 after 12h of HFNC application was an independent predictor of HFNC success. Although the AUROC of 24h ROX index is larger than that of 6h ROX index in present study, the 6h ROX index is a more suitable predictor of HFNC success considering both statistical and clinical significance. A ROX index greater than 5.55 at 6h after HFNC onset has a relatively low sensitivity (61.1%) and a relatively high specificity (84.6%). It is helpful for clinical patients to avoid delayed intubation, which has been proved to be unfavorable for prognosis. In present study, most of the intubations occurred on 2-7 days rather than within 24h HFNC treatment. The most likely reason is that, the patient population included in this study is non-ICU patients, excluding patients with respiratory failure who needed endotracheal intubation during initial oxygen therapy. This also implies that delayed intubation may be common in the real world of COVID-19, which may be related to poor prognosis.

This study had some mentionable limitations. First, this was a retrospective study. We did not predefine how to manage the HFNC. The transition to NPPV or IMV was decided by the attending physicians. Different physicians have different opinions on the point to switch to NPPV or IMV. However, this study can reflect on how the HFNC has been used in the real world among the COVID-19 patients. Second, the number of cases is not large enough. Only 105 patients were enrolled in this study. This is all COVID-19 patients who met our standard treated in two hospitals during this period. We hope to provide a true picture of HFNC treatment of COVID-19 for future reference when using HFNC to treat COVID-19 patients with hypoxic respiratory failure.

## Conclusion

Our study described the use of HFNC during the COVID-19 Outbreak and indicated that HFNC was an effective way of respiratory support in the treatment of severe COVID-19. Close monitoring of respiratory parameters is very important and will determine the next treatment strategy. The ROX index after 6h of HFNC application had good predictive capacity for HFNC outcomes.

## Data Availability

The datasets used and/or analyzed during the current study are available from the corresponding author on reasonable request.

## Abbreviations

COVID-19: 2019 novel coronavirus disease
HFNC: High-flow nasal cannula oxygen therapy
NPPV: Non-invasive positive pressure ventilation
NIV: Noninvasive ventilation
IMV: Invasive mechanical ventilation
ROX index: Respiratory rate-oxygenation index
